# Audiovisual integration and whole-brain networks in preterm and full-term neonates: A two-layer multiplex network perspective on structural and functional connectivity

**DOI:** 10.1162/IMAG.a.928

**Published:** 2025-10-17

**Authors:** Juan F. Quinones, Carsten Gießing, Axel Heep, Andrea Hildebrandt

**Affiliations:** Psychological Methods and Statistics, Department of Psychology, School of Medicine and Health Sciences, Carl von Ossietzky Universität Oldenburg, Oldenburg, Germany; Biological Psychology, Department of Psychology, School of Medicine and Health Sciences, Carl von Ossietzky Universität Oldenburg, Oldenburg, Germany; Research Center Neurosensory Science, Carl von Ossietzky Universität Oldenburg, Oldenburg, Germany; Cluster of Excellence Hearing4all, Carl von Ossietzky Universität Oldenburg, Oldenburg, Germany; Paediatrics, Department of Human Medicine, School of Medicine and Health Sciences, Carl von Ossietzky Universität Oldenburg, Oldenburg, Germany

**Keywords:** audiovisual integration (AVI), neonatal brain, multiplex network analysis, functional connectivity, structural connectivity, preterm birth

## Abstract

Audiovisual integration (AVI) is linked to the development of several cognitive abilities, rendering it a vehicle to better understand and anticipate a wide range of sequelae associated with preterm (PT) birth. In the present study, we aimed to complement the scarce literature on PT birth and AVI early in life, by investigating neonatal brain networks encompassing areas reported in the infant AVI literature. We used data from the Developing Human Connectome Project (http://www.developingconnectome.org/) to build two localized and one whole-brain connectomes from functional and structural connectivity data. Using graph analysis on a multiplex structural–functional brain network, we investigated the association between prematurity and (1) the edges present in functional and structural networks, (2) the integration and segregation properties of functional and structural networks, and (3) the inter-layer assortativity. We found substantial differences between PT and full-term (FT) neonates in the edges of the structural network at the whole-brain level and in one localized connectome. Across parcellation schemes, associations between prematurity and network efficiency were different, but similar inter-layer metrics were observed. In an exploratory analysis, we further showed lower functional connectivity strength in PT neonates. These findings are discussed in the light of perinatal brain developmental trajectories and deepen our understanding atypical AVI abilities in PT infants. More generally, the present work contributes to our understanding of whole-brain network development by investigating functional–structural coupling from a network neuroscience perspective.

## 1 Introduction

Currently, around 11% of all worldwide deliveries occur prematurely (i.e., before 37 completed weeks of pregnancy; Walani, 2020). Individuals born preterm (PT) are at risk of neurodevelopmental impairment, which is reflected in short- and long-term sequelae observed on biological, cognitive, and social levels (reviewed in Dean et al., 2021; Pascal et al., 2018; Pravia & Benny, 2020; Ream & Lehwald, 2018). Despite extensive research on auditory, visual, and cognitive sequelae, only few studies have focused on audiovisual (AV) integration (AVI). For instance, it has been shown that as compared with FT infants, PT infants between 3 and 7 months of age fail to detect AV synchrony (Pickens et al., 1994). Between 6 and 18 months of age, PT infants exhibit different patterns of discrimination (Berdasco-Muñoz et al., 2019) or processing (Imafuku et al., 2019) of AV material as compared with their FT counterparts. More recently, differences between PT and FT individuals between 12 and 24 months of age in terms of attentional biases toward face-speech stimuli were reported (Nakagawa et al., 2023).

The early onset of these differences is in line with a wealth of research suggesting that AVI abilities are in place from an early stage in life (e.g., Bahrick, 2001; Morrongiello et al., 1998) and are associated with cognitive development, for example, in terms of sustained attention (Curtindale et al., 2019), vocabulary size (Altvater-Mackensen & Grossmann, 2015), and affect discrimination (Flom & Bahrick, 2007). AVI is also related to social cognition (Mileva et al., 2018) and neuropsychological difficulties (Yang et al., 2020). As such, studying the neural correlates of AVI early in life and in the context of prematurity is a promising approach to deepen our understanding of PT birth sequelae and typical cognitive development. EEG and fNIRS studies on FT young infants have identified brain regions engaged in AV processing, including portions of the parietal, temporal, occipital, and frontal cortices (Altvater-Mackensen & Grossmann, 2016, 2018; Bristow et al., 2008; Hyde et al., 2011; Kopp, 2014; Kushnerenko et al., 2008; Reynolds et al., 2014, 2014). In addition, there is evidence of lateralized responses to AV stimuli (Altvater-Mackensen & Grossmann, 2016; Ujiie et al., 2018), suggesting the presence of functional asymmetries during early infancy. The reported brain areas largely overlap with those documented in children (Dick et al., 2010; Nath et al., 2011) and adults (Gao et al., 2022; Hickok et al., 2018; Zhou et al., 2020), and some of them are functionally and structurally linked shortly after birth (Quinones et al., 2022; Sours et al., 2017). More recently, Quinones et al. (2023) reported less developed white matter along such structural connections in PT neonates. However, whether these brain regions are functionally linked and underlay early AVI abilities remains unclear, in large part due to the difficulties of performing task-related functional neuroimaging on very young infants. With the current study, we aim to complement previous findings of structural connectivity (SC) among brain regions reported in the AVI literature, by investigating functional connectivity (FC) among them and how these two types of connectivity are related. Further, we investigate how network properties differ between PT and FT neonates.

SC and FC analyses allow the characterization of brain networks across the lifespan (Thomason et al., 2014; Fjell et al., 2017; Tsang et al., 2017). There is evidence that structural (Akazawa et al., 2016; Cohen et al., 2016; Feng et al., 2019) and functional (Fransson et al., 2007, 2009; Smith-Collins et al., 2015) architectures are partially or largely in place by the time of birth. A popular approach to brain connectivity is graph analysis, which provides several measures to characterize the topology of a network (Rubinov & Sporns, 2010). At term age, the brain structural network architecture is well established, but not necessarily mature. In fact, structural networks undergo an increase in segregation and integration during the prenatal and postnatal periods, respectively (Smyser et al., 2019; Zhao et al., 2019). Functional networks follow qualitatively similar trajectories, but considerable inter-network variability has been observed, with more mature functional networks comprising primary sensory and motor brain areas by the time of birth and during the neonatal period (Smyser et al., 2019). The effect of prematurity on brain connectivity has received considerable attention. Regarding SC, altered topology (Batalle et al., 2017) with atypical deep gray matter connectivity, increased cortico–cortical connectivity (Ball et al., 2014), and higher segregation (Sa de Almeida et al., 2021) have been reported in association with prematurity. Concerning FC, differences between FT and PT neonates are more apparent in sub-cortical connections (Brenner et al., 2021), although overall differences in various graph measures, for example, clustering coefficient, have been reported at term equivalent age (TAE; Smyser et al., 2019).

The relationship between SC and FC is not yet fully understood (Hahn et al., 2019). For instance, while commonly found resting-state networks reflect the underlying structure of major white mater tracts (van den Heuvel et al., 2009), FC among brain areas can occur in the absence of direct structural connections among them. In general, however, there is ample evidence of correspondence (*r* = 0.18–0.82) between SC and FC (Skudlarski et al., 2008; Straathof et al., 2019), suggesting that functional properties may be successfully predicted from SC data (Neudorf et al., 2022). A powerful tool to characterize the organization and relationships across different domains in complex data systems is multiplex networks (Interdonato et al., 2020), which take multilayer features into account when describing multiple subsystems that pertain to a complex general system (Kivela et al., 2014). Multiplex networks provide a more realistic representation of the heterogeneous relations that characterize a network system (Interdonato et al., 2020). In neuroscience, functional and structural networks have been conceptualized as different layers within a system (e.g., Lim et al., 2019). Investigating multiplex networks together with graph theory metrics can yield additional information to further understand the correspondence between SC and FC. For example, a system’s robustness (Garas, 2016) can be characterized by assortativity metrics, which indicate whether network components have similar properties in different layers of the multiplex network. To our knowledge, no study has investigated coupling between SC and FC networks in FT and PT neonates, but studies on adults have shown strong assortativity in certain networks (e.g., Lim et al., 2019).

The present study aims to investigate whether prematurity is associated with the properties of neonatal structural and functional networks involving brain regions that have been reported in the literature to be involved in AVI. In addition, we explore for the first time the relationship between prematurity and the overlap between these networks using inter-layer metrics. The initial analyses were preregistered (https://osf.io/5px9e/). We postulated two confirmatory (one-tailed tests) hypotheses and an exploratory one. First, we expected that FT and PT neonates would differ in terms of the connections (i.e., edges) present in the structural and functional networks comprising brain regions involved in AVI. Second, we expected network segregation and integration properties, as indicated by local and global efficiency, respectively, to be positively associated with gestational age (GA) at birth. Third, we aimed to explore whether prematurity is associated with the correspondence (multilayer edge overlap and node assortativity) between functional and structural brain networks. To assess the generalizability and specificity of our findings, in an exploratory non-preregistered analysis, we computed additional network metrics and reproduced all analyses on two additional parcellation schemes derived from a recently developed neonatal functional parcellation (Myers et al., 2024).

## 2 Methods

A detailed description of the methodological procedures can also be found in the preregistration form that accompanies this manuscript (see https://osf.io/5px9e/).

### 2.1 Participants

The datasets were obtained from the third data release of the dHCP (http://www.developingconnectome.org/project/). This includes 886 datasets from 783 neonates. The study was approved by the UK Health Research Authority (Research Ethics Committee reference number: 14/LO/1169) and parental written consent was obtained. Recruitment took place at the Evelina Newborn Imaging Centre, St Thomas’ Hospital in London, UK. Main inclusion criteria were (1) pregnant woman with fetal age estimated from the last menstrual period between 22 and 44 weeks and (2) living infants between 23 and 44 weeks of GA. Main exclusion criteria were (1) contraindication to undergo MRI scanning in the mother of the infant, (2) expert medical advice against MRI due to health conditions in PT infants, and (3) failed communication regarding study procedures and consent. We selected datasets of singleton infants imaged at TEA (37–44 weeks of Post Menstrual Age—PMA) with available structural MRI, dMRI, and rs-fMRI scans and whose radiology score was 4 or less. Radiology scores range from 1 (optimal scan quality) to 5 (possible clinical or analysis implications). The resulting PT neonates were matched to FT infants using propensity score matching (Austin, 2011) to reduce covariate imbalance in PMA at scan and reported biological sex of the infants. The final sample consisted of 59 FT and 59 PT neonates. Further information is summarized in Table 1.

**Table 1.** Demographic information of the study sample.

Subgroup	Assigned sex at birth	*N*	GA at birth (weeks)	PMA at scan (weeks)	Birth weight (kg)
FT	Female	18	39.56 (1.32)	40.94 (1.66)	2.99 (0.5)
FT	Male	41	39.37 (1.14)	40.64 (1.8)	3.36 (0.43)
PT	Female	13	34.27 (2.14)	39.99 (2.4)	2.22 (0.76)
PT	Male	22	34.85 (1.73)	40.28 (1.82)	2.41 (0.55)
vpt	Female	5	29.6 (0.63)	41.29 (1.61)	1.22 (0.14)
vPT	Male	10	28.99 (0.75)	40.87 (1.83)	1.13 (0.21)
ePT	Female	5	25.23 (1.18)	41.54 (1.11)	0.78 (0.13)
ePT	Male	4	26.25 (1.26)	41.25 (1.64)	0.78 (0.2)

FT—full-term; PT—preterm; vPT—very preterm; ePT—extremely preterm; GA—gestational age at birth; PMA—postmenstrual age at scan. Standard deviation is provided within parentheses.

### 2.2 MRI acquisition

MRI data were acquired using a 3T Philips Achieva MRI scanner with a dedicated neonatal imaging system (Hughes et al., 2017). In all but six cases, infants were scanned during natural sleep. The total scanning time was 63 minutes. For a full description of the acquisition procedures, refer to the scientific publications on preprocessing pipelines of the dHCP (Bastiani et al., 2019; Bozek et al., 2018; Fitzgibbon et al., 2020; Makropoulos et al., 2018).

#### 2.2.1 Structural MRI (sMRI)

T2w (TR = 12000 ms, TE = 156 ms) and T1w (TR = 4795 ms, TE = 8.7 ms) inversion recovery multi-slice fast spin-echo images were acquired with in-plane resolution of 0.8 x 0.8 mm^2^ in sagittal and axial slice stacks. Slice thickness was 1.6 mm. The slices overlapped by 0.74 mm for T1w sagittal stacks and otherwise 0.8 mm. 3D MPRAGE (TR = 11 ms, TE = 4.6 ms) images were additionally acquired with a 0.8 mm isotropic resolution.

#### 2.2.2 Resting-state fMRI (rs-fMRI)

A dedicated high temporal resolution fMRI sequence was developed for neonates. It consisted of 9 acceleration-factor multiband echo-planar imaging (TR = 392 ms, TE = 38 ms) acquired in 15 minutes and resulting in 2300 volumes with an isotropic resolution of 2.15 mm.

#### 2.2.3 Diffusion MRI (dMRI)

dMRI data were acquired using an optimized set of directions on 4 shells (b-values: 0, 400, 1000, and 1600) and divided into 4 subsets according to each phase encoding direction. TR and TE were set to 3800 ms and 90 ms, respectively. A multiband acceleration factor of 4 was used. In-plane resolution was 1.5 x 1.5 mm^2^ and slice thickness was 3 mm with a 1.5 mm overlap. The complete dMRI dataset contained 300 volumes.

### 2.3 MRI preprocessing

MRI data already preprocessed by the dHCP scientific collaborators were used for the present work. The preprocessing pipelines were specifically developed to tackle challenges posed by neonatal MRI and are publicly available. For complete information about sMRI, dMRI, and rs-fMRI, we refer to the dHCP publications (Bastiani et al., 2019; Bozek et al., 2018; Fitzgibbon et al., 2020; Makropoulos et al., 2018).

#### 2.3.1 Structural preprocessing pipeline

sMRI data were in-plane and across-slices motion corrected. Orthogonal slice stacks (orthogonal and axial) were integrated to reconstruct anatomical images via a superresolution algorithm (Bastiani et al., 2019). Then, data were preprocessed in four major stages: (1) registration; (2) segmentation, including bias correction, brain extraction, segmentation, and labeling of the T2w volume and anonymization; (3) surface extraction yielded white matter, mid-thickness, and pial surfaces, as well as curvature, cortical thickness, sulcal depth, and myelin maps; and (4) surfaces were non-linearly registered to the extended neonatal surface template.

#### 2.3.2 rs-fMRI

The rs-fMRI preprocessing pipeline consisted of four major stages: (1) preparation of field maps to correct for susceptibility distortions; (2) registration, which outputs warp and affine transformation files between rs-fMRI data and the 40-week template from the dHCP volumetric atlas and structural space, respectively; (3) data were corrected for motion and susceptibility distortions; and (4) data were high-pass filtered with 150 seconds as cutoff and denoised by means of ICA. As an additional step, we applied a low-pass filter with a cutoff value of 0.1 Hz.

#### 2.3.3 dMRI

We analyzed dMRI data resulting from the EDDY preprocessing pipeline, which consisted of six main stages: (1) 4D raw data were reorganized based on the extent of intra-volume motion in non-diffusion-weighted (b0) volume pairs for each phase encoding direction; (2) field maps were estimated to correct for susceptibility-induced distortions; (3) data were corrected for between- and within-volume motion, susceptibility, signal drop-out, eddy currents, and motion-by-susceptibility interactions; (4) a super-resolution algorithm was used to integrate overlapping slices and achieve a 1.5 mm isotropic resolution; (5) a tensor model was fit to the data and distributions of diffusion parameters were estimated at each voxel; and (6) transformation files between diffusion and the T2w volume and the 40-week template from the dHCP volumetric atlas were generated.

### 2.4 Connectome formation

#### 2.4.1 Regions of interest

**Fig. 1.**
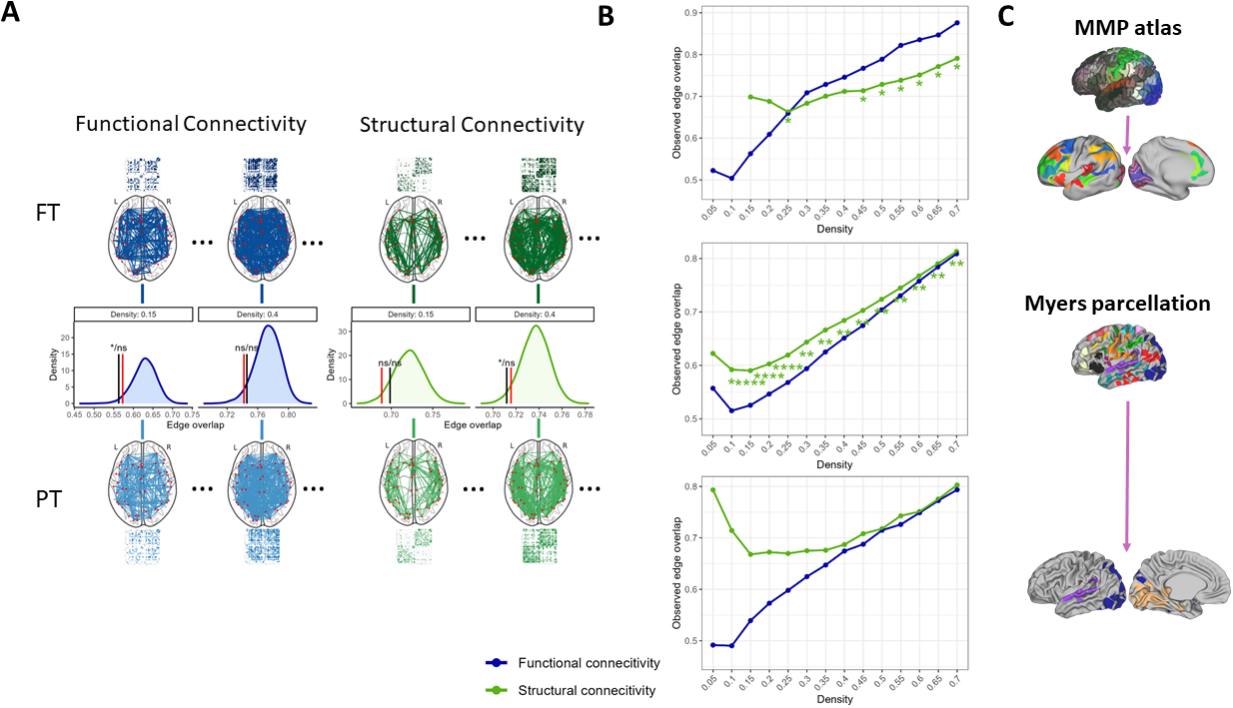
(A) Summary of the procedure followed to test the first hypothesis: For each of the three parcellation schemes (Panel C), functional and structural connectomes are generated at different density levels (0.15 and 0.4 as example) for FT and PT neonates. For each connectivity modality independently and density level, empirical edge overlap scores are computed between group-level (FT and PT) connectomes and evaluated relative to a simulated null distribution of the same scores. The black and red vertical lines in the density plots represent the *p-*value of the empirical edge overlap score before and after correcting for multiple comparison, respectively. (B) Summary of empirical edge overlap scores when comparing FT against PT neonates for structural and functional connectomes independently at each density level (Within-modality, between-groups). The * indicates statistical significance according to the procedure in B. * (*p* < .05) after adjusting for multiple comparison across density levels.

EEG, fNIRS, and fMRI studies on infants have consistently reported a widespread of brain areas engaged in AV processing, such as the inferior frontal gyrus (Watanabe et al., 2013), temporal cortices (Ujiie et al., 2021; Werchan et al., 2018), the superior temporal sulcus (Bristow et al., 2008; Ujiie et al., 2018), visual areas (Emberson et al., 2015, 2017), the inferior parietal sulcus (Sours et al., 2017), and frontal cortices (Altvater-Mackensen & Grossmann, 2018 Hyde et al., 2011; Kopp, 2014; Kushnerenko et al., 2008). In this study, regions of interest (ROIs) were created by merging brain parcels extracted from the Multi-Modal Parcellation atlas, which in total includes 180 parcels per brain hemisphere (MMP atlas; Glasser et al., 2016). This resulted in 25 cortical ROIs per hemisphere located in the occipital, temporal, parietal, and frontal lobes (see Fig. 1). In addition, we included the superior colliculi as an ROI, which was extracted from a volumetric atlas (Brainstem Navigator atlas; García-Gomar et al., 2019). A complete list with the ROIs is given in Table A.1 in the Supplementary Material section. Cortical ROIs were transformed to individual structural space by following the procedure described in Quinones et al. (2023). In brief, the Multimodal Surface Matching (MSM; Robinson et al., 2014, 2018) and the connectome workbench (Marcus et al., 2011) software were used to perform surface-based registrations. Regions of interest were transformed to volumetric files using white matter and mid-thickness layers to perform SC and FC analyses, respectively. This procedure requires the label files to be in native space. Individual surface ROIs extracted from the label files can be mapped to a participant’s surface (e.g., white matter) using the FSL function *surf2surf* and then transformed to volumetric files using the FSL function *surf2volume*. Because the superior colliculi were originally in volumetric format, it was directly transformed to individual structural space by means of linear and non-linear registrations using FSL (https://fsl.fmrib.ox.ac.uk/fsl/fslwiki/).

#### 2.4.2 Functional connectivity

Preprocessed rs-fMRI datasets were transformed to individual structural space using FSL (https://fsl.fmrib.ox.ac.uk/fsl/fslwiki/) and MIRTK (https://mirtk.github.io/) software. The transformation files between structural and functional spaces are provided as part of the data releases from the dHCP. Time series were extracted from each participant’s rs-fMRI data using the Nibabel v. 3.2.0 and Nilearn v. 0.7.1 libraries on Python-3.7.4 and a brain template in native space containing all ROIs (see Section 2.5.1). Each time series corresponds to the average within a given ROI as defined in Section 2.5.1. Next, functional connectomes were created by computing Pearson correlations for each time series file, resulting in three correlation matrices for every participant. We used R (R Core Team, 2022) version 4.2.1 to transform negative correlations by computing their absolute value (Centeno et al., 2022; Hallquist & Hillary, 2019) and to transform them to *z*-scores using Fisher’s *r-*to-*z* transformation.

#### 2.4.3 Structural connectivity

We used the BEDPOSTX functionality in FSL v. 6.0.4 with GPU support (bedpostx_gpu) on preprocessed dMRI data with the same parameters as in the dHCP EDDY pipeline. We then performed probabilistic tractography with GPU support in network mode on FSL v. 6.0.4 to generate a connectivity matrix for each brain hemisphere independently and together. We included ROIs as described in Section 2.5.1 pial masks as stop criteria. Default values for number of samples (5000), number of steps (2000), and step length (0.5 mm) were kept. These structural connectomes were adjusted for path length and ROI size by dividing the average (computed across both tracing directions) streamline count in each matrix entry by the average size of the two ROIs sharing that connection.

### 2.5 Graph processing

#### 2.5.1 Thresholding

Thresholding and subsequent processing were carried out on R (R Core Team, 2022). Two different thresholding procedures were applied: (1) To address the first research question (assessment of edge overlap between connectomes from FT and PT neonates), we used consistency-based thresholding (Roberts et al., 2017) for both connectivity modalities (structural and functional) and neonates groups (FT and PT) independently. Consistency thresholding is useful when performing group-wise comparisons and has been shown to outperform other thresholding approaches (Buchanan et al., 2020). It requires the computation of a coefficient of variation across participants (Roberts et al., 2017). This coefficient is then used to retain a given set of the most consistent connections across participants (Roberts et al., 2017). (2) To address the remaining research questions (assessment of segregation, integration, and multiplex properties), we applied proportional (i.e., density) thresholding (Bullmore & Bassett, 2011; Rubinov & Sporns, 2010) for each participant and modality independently. This approach retains a fixed proportion of the strongest connections to guarantee connectomes of equal density. Both thresholding procedures were used to generate 14 connectomes with equally graded densities ranging from 5% to 70% of the most reliable edges. Finally, all connectomes were binarized.

#### 2.5.2 Network metrics

Research question 1: For each connectivity modality independently, consistency-thresholded connectomes of the same density were compared between the FT and PT groups (within-modality, between-groups). We used the muxViz R package (De Domenico et al., 2015) to compute the extent of edge correspondence (i.e., edge overlap) between two connectomes. This metric ranges between 0 and 1 for null and complete overlap, respectively.

Research question 2: Using the igraph package (Csárdi & Nepusz, 2006), we then computed global and local efficiency graph metrics on the functional and structural proportionally thresholded connectomes. Global efficiency is defined as the average of inverse distances (shortest path connecting two vertices) between all pairs of vertices (Csárdi & Nepusz, 2006). Nodal local efficiency is computed as the average global efficiency of the subgraph formed by the node’s neighbors and excluding the node itself. We refer to local efficiency as the arithmetic mean of all nodal local efficiency scores (Csárdi & Nepusz, 2006).

Research question 3: Inter-layer (multiplex) metrics of edge overlap and node assortativity were computed for each participant across proportionally thresholded connectomes of different modality using the muxViz R package (De Domenico et al., 2015). Within a single layer, nodes can be predominantly connected to nodes of similar (assortative correlation) or dissimilar (dissortative correlation) degree (Nicosia & Latora, 2015). This can be extended to multiplex networks by computing degree–degree correlations between sequences of different network layers. Node assortativity, therefore, indicates the similarity of node degree structure between two different network layers. We measured node assortativity by computing the Spearman and Pearson correlation coefficients between nodal degrees from structural and functional networks (Noldus & Van Mieghem, 2015).

### 2.6 Statistical analysis

To address the first hypothesis (confirmatory), we performed a series of permutation tests for testing group differences. An empirical edge overlap metric was computed between FT and PT connectomes for each density level and for each connectivity modality independently. That is, comparisons were carried out within connectivity modality and between groups (e.g., structural connectome from FT and PT groups). Neonates were then randomly assigned to either group and edge overlap metrics were computed again. This process was repeated 10.000 times to simulate a null distribution and a one-tail test was used. Differences between FT and PT groups were regarded as significant if the probability of obtaining the empirical edge overlap score was lower than the 0.05 cutoff in the null distribution.

To address the second hypothesis (confirmatory), we estimated four multilevel regression models with brain hemisphere as within-person factor, density levels as a nested factor, and interaction effects between GA at birth and brain hemisphere. Global and local efficiency in SC and FC networks were the dependent variables in four separate models. The resulting effects indicate the association between GA at birth and brain hemisphere (and their interaction) with segregation and integration properties in both connectomes at different density levels.

To address our exploratory hypothesis, this regression analysis was repeated but with inter-layer metrics as dependent variables instead (i.e., structural–functional edge overlap and node assortativity). The *p*-values resulting from each hypothesis test were corrected for multiple comparison using the Benjamini–Hochberg method (Benjamini & Hochberg, 1995). Outliers were defined as values exceeding 1.5 times the lower or upper boundaries of the interquartile range and then removed from segregation, integration, and multi-layer metrics before including them in regression models.

### 2.7 Exploratory analysis

#### 2.7.1 Area under the curve and connectivity strength

Topological properties can vary as a function of the density of an individual network (Ginestet et al., 2011), thus requiring to disentangle connectivity strength from network topology. Investigating network properties across a range of network densities reduces the arbitrariness of selecting a single cutoff threshold from which conclusions are derived (Buchanan et al., 2020; Jalili, 2016). By doing so, networks’ topologies can be compared irrespective of their connectivity strength (Ginestet et al., 2011). Despite selecting a wide density range and a short increasing step, this approach may neglect topological differences that are visible at omitted density levels. As an exploratory non-preregistered analysis, we aggregated global and local efficiency scores individually across density levels by computing the area under the curve. The aggregated scores were included in single multilevel regression models with GA at birth, brain hemisphere (within factor), and their interaction as predictors. Moreover, because connectivity strength reflected on weighted networks may be informative about brain processes (Ginestet et al., 2011), we investigated differences between FT and PT neonates. Individual structural and functional connectomes (after removing negative correlations) were averaged, resulting in two connectivity strength scores per participant. Then, we ran a two-tailed *t*-test for each connectivity modality independently.

#### 2.7.2 Neonatal parcellation

The selection of a parcellation scheme from which network nodes are selected is critical in graph analysis (Hallquist & Hillary, 2019; Rubinov & Sporns, 2010). In the preregistered analysis, we used the MMP atlas (Glasser et al., 2016) because of its multimodal nature, high ROI resolution, and compatibility with our previous work (Quinones et al., 2022, 2023). However, neonatal-dedicated surface templates may provide a better fit to neonatal data (Myers et al., 2024). We repeated all analyses described thus far using a neonatal parcellation derived from FC patterns (Myers et al., 2024), hereafter referred to as the Myers parcellation. The complete parcellation divides the cortex into 283 parcels and assigns them a functional network identity. We built connectomes including the entire parcellation and a subset of the parcels, which are assigned to visual and auditory networks (number of parcels = 85).

## 3 Results

### 3.1 Preterm birth and network edge overlap

Figure 1 depicts the parcels included in the different parcellation schemes for the present study (panel C), as well as the permutation procedure conducted to address the first hypothesis (panel A). Across parcellation schemes, the empirical edge overlap scores between FT and PT neonates range from 0.5 to 0.8 approximately (see panel B), indicating a between-group edge overlap of around 50% even at high sparsity levels. Overlap scores show an association with density levels for both connectivity modalities, such that higher density levels yield larger edge overlap scores and, conversely, smaller edge overlap scores were found at lower density levels. The variation in edge overlap scores across density levels is larger for the functional than the structural connectome. The results from the permutation tests show that for all parcellation schemes, none of the edge overlap score differences for FC is significant, thus indicating that FT and PT neonates do not differ in terms of the edges present in the functional connectomes.

By contrast, the differences in edge overlap scores are significant for the structural connectomes at density levels larger than 0.4 for the MMP atlas, and across all density levels for the full Myers parcellation. This indicates that as opposed to FC, there are substantial differences between FT and PT neonates in the network architecture extracted from these two parcellation schemes, but none when contemplating parcels that belong to visual and auditory networks exclusively. Of note, the results from density levels 0.05 and 0.10 pertaining to the structural connectome derived from the MMP atlas were omitted because the simulated null distributions showed critical deviations from normality (see Fig. A.1 in the Supplementary Material section). A detailed justification is provided in Section 4.6.

### 3.2 Preterm birth and network properties

**Fig. 2.**
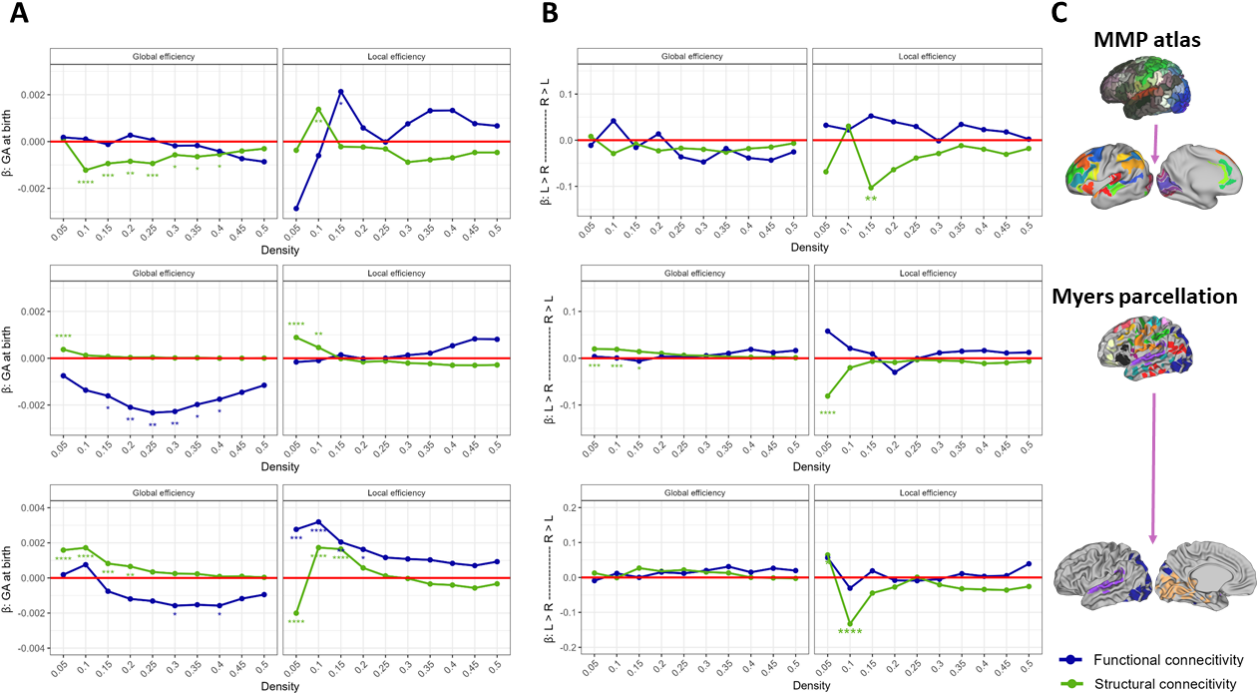
Regression parameters of global and local efficiency on GA at birth in structural and functional connectomes extracted from three parcellation schemes (panel C). Non-standardized regression slope estimates for GA at birth (panel A) and brain hemisphere (panel B) are shown across network density levels. Estimates above and below the red horizontal line indicate positive and negative associations, respectively. Significance levels after adjusting for multiple comparison: * (*p* < .05), ** (*p* < .01), *** (*p* < .001), **** (*p* < .0001).

To test the association between PT birth and network properties in functional and structural connectomes, we used multilevel regression models with brain hemisphere as a within-person factor and density levels as a nested factor. We hypothesized that GA at birth is positively associated with integration and segregation properties in the reconstructed structural and functional networks. Panels A and B in Figure 2 depict the main effects of GA at birth and brain hemisphere on integration and segregation network properties for the three parcellation schemes included in this study. Global efficiency in the SC network comprising ROIs from the MMP atlas and the visual and auditory networks from the Myers parcellation is predicted by GA at birth at high sparsity levels, but the effects are of opposite direction, suggesting contradictory associations with prematurity. Substantial effects of local efficiency were estimated across low-density networks including the auditory and visual parcels from the Myers parcellation and describe negative associations with prematurity for FC and both negative a positive association for SC. Regarding the complete Myers parcellation, our findings reveal a significant positive effect of GA at birth on SC local and global efficiencies at high-sparsity levels only, and a more consistent negative effect on FC global efficiency.

In line with our prediction, interhemispheric differences were generally absent for the MMP atlas but also when investigating the complete Myers parcellation and its visual and auditory components (panel B in Fig. 2), with only a few significant effects for SC at high-sparsity levels. The corresponding interaction effects are shown in Figure A.2 in the Supplementary Material section. Results from regression analysis at network density levels higher than 0.5 were considered misleading because the large proportion of edges present in the network reduced inter-individual differences, therefore, affecting distributional properties required for the regression models (see Fig. A.3 in the Supplementary Material section). These results are consequently excluded from the present study. For more details, see Section 4.6.

### 3.3 Preterm birth and structure–function correspondence

**Fig. 3.**
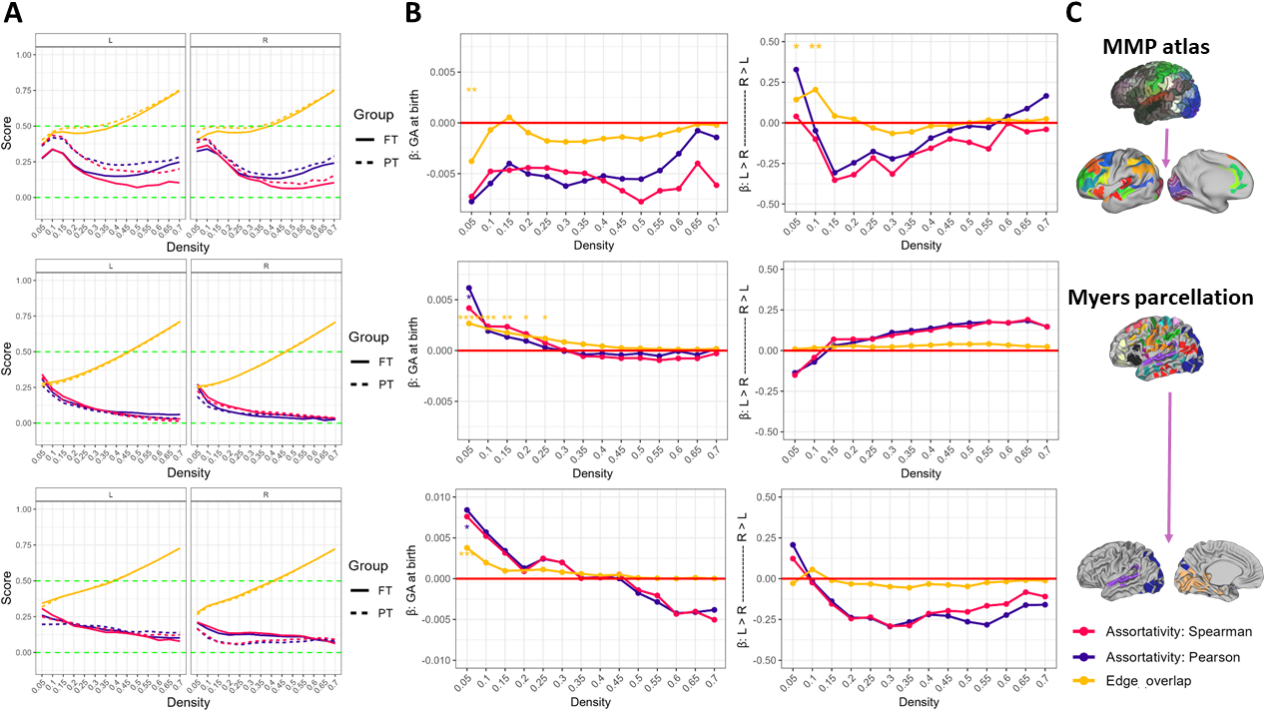
Group-wise mean scores of three interlayer metrics between structural and functional connectomes across density levels are depicted for three parcellation schemes (Panel C) and each brain hemisphere (Panel A). Regression parameters of these inter-layer metrics on GA at birth and brain hemisphere are shown in Panel B: Left column: non-standardized regression slope estimates for GA at birth. Right column: non-standardized regression slope estimates for brain hemisphere. Estimates above and below the red horizontal line indicate positive and negative associations, respectively. Significance levels after adjusting for multiple comparison: * (*p* < .05), ** (*p* < .01).

To explore the association between prematurity and the correspondence between structural and functional networks, we computed three inter-layer metrics (i.e., edge overlap scores and two measures of node assortativity). Figure 3 depicts mean scores across density levels and results from a regression analysis with GA at birth and brain hemisphere as predictors nested within network density levels. Edge overlap scores across parcellation schemes range between 0.25 and 0.75 approximately for FT and PT neonates and increase with network density (Panel A). Assortativity measures take on small-to-medium positive values, which suggest similar rather than different rank order and degree distributions between structural and functional connectomes in both groups. In general, regression coefficients corresponding to main (Panel B) and interaction effects (Fig. A.2 in the Supplementary Material section) for the MMP atlas and the visual and auditory networks of the Myers parcellation are not significant. The complete Myers parcellation shows significant positive effects more consistently across high-sparsity levels for edge overlap, indicating that higher scores of GA at birth predict higher edge correspondence between whole-brain functional and structural networks. Interhemispheric differences are generally absent with only a few significant effects on edge overlap at high-sparsity levels for the MMP atlas, hinting a rightward asymmetry. The absence of significant regression coefficients for both measures of assortativity (i.e., Spearman and Pearson correlation), as well as similar curve shapes across network density levels for all three parcellation schemes, suggests no extreme deviations between the two correlation methods.

### 3.4 Area under the curve and connectivity strength

**Fig. 4.**
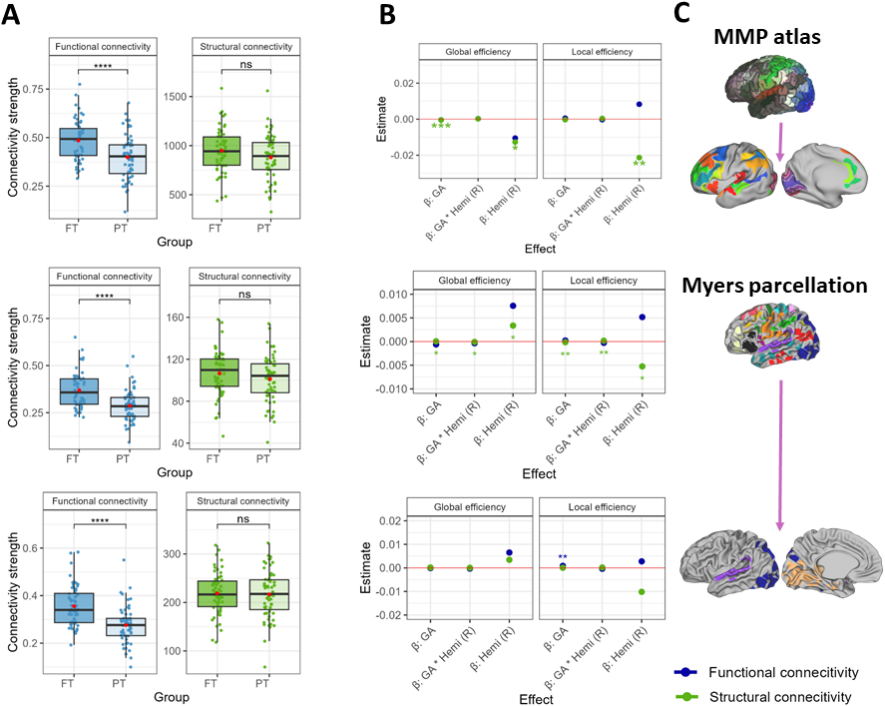
(A) Between-group comparison of functional and structural connectivity strength. (B) Regression coefficients for main and interaction effects of GA at birth and brain hemisphere on local and global efficiency scores aggregated across density levels for functional and structural connectomes (area under the curve). (C) Three parcellation schemes used in the present study to compute global and local efficiency. Significance levels after adjusting for multiple comparison: ns—non-significant; * (*p* < .05), ** (*p* < .01), *** (*p* < .001), **** (*p* < .0001). GA—gestational age.

To assess between-group differences in connectivity strength, we performed an exploratory non-preregistered analysis. Results from *t*-tests for each connectivity modality are shown in Figure 4. We found substantial differences in FC strength but not in SC strength across all three parcellation schemes. The additional regression analysis with global and local efficiency scores aggregated across density levels (Panel B) revealed a positive effect of GA at birth on local efficiency in the FC connectome extracted from the visual and auditory networks of the Myers parcellation. Concerning the connectome extracted from the MMP atlas, a statistically significant negative regression weight for global efficiency in the structural network suggests that by TEA, prematurity is associated with larger scores. For both efficiency metrics, our exploratory analysis indicates a leftward asymmetry in the structural network. By contrast, when considering the full Myers parcellation, the positive effect of GA at birth suggests an advantage in SC global efficiency for FT participants. This association is more pronounced for the right hemisphere. The opposite pattern was found for local efficiency in the SC network, as lower GA at birth predicts higher local efficiency and this association is more pronounced in the left brain hemisphere.

## 4 Discussion

PT individuals are at risk of a displaying wide array of physical, cognitive, and social sequelae. The developmental links of AVI with several higher cognitive abilities hint its role in better understanding and anticipating some of these sequelae. However, AVI has been only scarcely investigated in relation to prematurity early in life (e.g., Berdasco-Muñoz et al., 2019; Imafuku et al., 2019). In the absence of functional neuroimaging data on very young infants during AV tasks, the present work sought to investigate the association between prematurity and structural and functional networks encompassing brain regions consistently reported in the AVI literature. We contrasted our findings with those obtained from a whole-brain neonatal parcellation and its visual and auditory subnetworks.

### 4.1 Network edge overlap

During the third trimester of pregnancy, the brain’s structural architecture undergoes rapid maturation. For example, exuberant axon growth and oligodendrocyte formation take place during this time (Back, 2017; Dubois et al., 2014; Qiu et al., 2015). The abrupt interruption of pregnancy may alter these and other ongoing processes (e.g., proper myelination in other brain regions) and lead to atypical structural and functional brain connectivity (Smyser et al., 2019). Although some aspects of structural and functional brain networks at TAE seem unaffected in PT neonates (e.g., Ball et al., 2014; Eyre et al., 2021), some others have been linked to prematurity, for instance, short-range cortical connections and interhemispheric connections (Batalle et al., 2017). We, therefore, hypothesized that a largely distributed network comprising short- and long-range connections among brain areas engaged in AVI would exhibit architectural differences between FT and PT neonates at TAE.

Our findings showed that the reconstructed functional and structural networks of FT and PT neonates overlap considerably. While edge overlap scores are seemingly dependent on network density (i.e., overlapping edges are more likely to be found in networks with more edges), we found considerable overlap even on the lowest density levels included in the analysis. This holds true for the three parcellation schemes included in this study and suggests that even under sparsity conditions, FT and PT groups share between approximately 50% and 80% of the most consistent connections in functional and structural networks. Permutation-based tests showed that only edges present in structural connectomes were significantly different between groups. In particular, between-group differences were observed mostly at higher density levels (>0.4) in the connectome derived from the MMP atlas, across all density levels in complete Myers parcellation, but were absent when considering its visual and auditory components only.

Differences in network edge structure pertaining to structural connectivity only may reflect the developmental order of structural and functional brain networks during early development, as structural networks develop before and so “pave” the way for the functional ones (Ciarrusta et al., 2022; Duerden et al., 2019; Hagmann et al., 2010; Zhao et al., 2019). More advanced consolidation of structural networks by birth time reveals between-group differences that may be later reflected in FC. This is in line with the superior stability of the structural connectome as compared with the functional one during the perinatal period (Ciarrusta et al., 2022) and with patterns of white matter microstructure development. Quinones et al. (2023) reported that differences between FT and PT neonates in the developmental order of brain pathways are more apparent when employing metrics strongly associated with maturation. Larger variation of between-group edge correspondence across density levels for the functional network supports the idea that structural networks are more consolidated. Overall, our findings are in line with our hypothesis only in part. We showed that there is considerable between-group overlap in structural and functional networks comprising brain regions broadly reported in the infant AVI literature. Despite this, both groups substantially differ in the edges included in the structural network at higher density levels. That is, substantial between-group differences are apparent when less consistent connections across single groups are included in the connectomes. Whether this is a reflection of between-group differences in the structural connections undergoing maturation after a more stable fingerprint has been formed (Ciarrusta et al., 2022), or reveals that PT individuals may recruit other connections within the proposed network, as has been documented for particular abilities, such as language (Ream & Lehwald, 2018), cannot be answered with the data at hand. In addition, the absence of substantial between-group differences when considering parcels assigned to visual and auditory networks only speaks against an effect of prematurity on the edge network architecture among these brain regions.

### 4.2 AVI network integration and segregation

Numerous studies have reported differences in network integration and segregation properties between FT and PT individuals (Batalle et al., 2017; Bouyssi-Kobar et al., 2019; Smyser et al., 2019; Zhao et al., 2019). For example, cumulative data suggest that as compared with PT neonates, structural networks in FT neonates exhibit increased segregation and integration capacities (Zhao et al., 2019). Similarly, smaller clustering coefficients and reduced modularity are observed in PT neonates at term (Smyser et al., 2019). Because an increase in integration and segregation capacities in functional and structural networks is expected during the prenatal period (Batalle et al., 2017; Brenner et al., 2021; Zhao et al., 2019), we hypothesized that global and local efficiency scores computed on the reconstructed networks would be larger for FT neonates.

Our findings across parcellation schemes are heterogeneous and align only in part with this expectation. When considering the whole-brain Myers parcellation, significant regression weights are almost entirely restricted to FC global efficiency across mid-range network density and describe an advantage for PT individuals by TEA. The connectome derived from the MMP atlas yielded mostly negative significant regression coefficients for global efficiency in the structural network at low-to-mid density levels only. This suggests that prematurity is associated with higher integration capacity in the proposed AVI structural network by TEA. The hypothetical model of topological development by Zhao et al. (2019) posits an increase in integration capacity following birth, which would account for the observed negative effects. However, this is in contradiction with the findings from the parcellation scheme including visual and auditory networks only, according to which prematurity is associated with lesser SC global efficiency and FC local efficiency. It has been suggested that before the typical time of delivery, long-range connections increase at a faster rate than short-range connections (Batalle et al., 2017). Based on our findings, this suggests that although preterm neonates experience a sudden interruption in in-utero maturation, they may be less disadvantaged in developing long-range connectivity. This, in turn, could support a marked increase in network integration capacities during the neonatal period, provided they receive sufficient experiential input. However, shorter-range cortico–cortical connections (Batalle et al., 2017), as those included in the reduced Myers parcellation, are more susceptible to be affected by prematurity, thus showing an advantage for FT neonates.

Whether the advantageous SC global efficiency for PT neonates extracted from the MMP atlas corresponds to accelerated development, as has been documented for some structural pathways using diffusion-based metrics (Ream & Lehwald, 2018), or represents atypically increased integration capacities that may contribute to differences in AV-processing abilities (e.g., Nakagawa et al., 2023), requires further research. For instance, within the fetal programming framework, overstimulation (e.g., under intensive care conditions) is one of the factors accounting for neurodevelopmental sequelae in PT population (Fernandes et al., 2022; Ream & Lehwald, 2018). Finally, when considering the reduced Myers parcellation, we found substantial positive regression weights across low-to-mid network densities. Because sensory networks develop first (Smyser et al., 2019), this finding suggests an advantage for FT neonates in terms of FC local efficiency among brain areas assigned to auditory and visual networks.

### 4.3 Multiplex network correspondence

Brain networks have increasingly gained attention over the past decades as a fruitful approach to understand “mental function” (Pessoa, 2014). These networks are generally categorized as functional or structural, both of which have been related to cognition (Hahn et al., 2019; Park & Friston, 2013; Pessoa, 2014). A key question relates to the association between these networks and its change over the lifespan. Most studies on the association between SC and FC networks have focused on adulthood (Fjell et al., 2017; Hahn et al., 2019; Straathof et al., 2019), despite the growing interest on brain networks during early infancy (Batalle et al., 2017; Brenner et al., 2021; Smyser et al., 2019; van den Heuvel et al., 2015; Zhao et al., 2019). In the present work, we reconstructed neonatal functional and structural networks encompassing the entire brain and brain regions involved in AVI and explored their correspondence. To our knowledge, this is the first study to explore the relationship between neonatal SC and FC from a multilayer graph perspective and its association with PT birth.

For all the parcellation schemes included in this study, our findings showed variable degrees of edge overlap between the reconstructed functional and structural networks across density levels. As compared with edge overlap scores computed between PT and FT groups for a single connectivity modality, SC–FC edge overlap scores were on average smaller. This indicates larger intra- than inter-modality edge correspondence, and is potentially grounded on strong genetic control (Burger et al., 2022) and different rates of maturation in SC and FC. As noted before, although developmental trajectories are similar, the structural connectome remains ahead the functional one (Zhao et al., 2019). In addition, it has been shown that SC–FC coupling increases with age (Hagmann et al., 2010), predicting lesser correspondence between SC and FC shortly after birth. However, the different thresholding approaches followed for the first and third research questions may have induced statistical artifacts that prevent a direct comparison of overlap scores between- and within-participants. Assortativity measures varied from small-to-moderate effects but were positive in all cases. To our knowledge this is the first estimation of SC–FC coupling in terms of node-to-node degree correspondence at term age. The findings indicate that on average, both networks tend to be similar in this regard, which can be interpreted as increased robustness of the system (i.e., increased layer interdependency; Reis et al., 2014), by mitigating the abrupt nature of percolation transitions (Lim et al., 2019). In practice, higher robustness may explain why brain function can be retained unless large-scale damage takes place (Lim et al., 2019). Overall, these findings are in line with the literature advocating for a positive SC–FC coupling during the neonatal period (van den Heuvel et al., 2015) and later in life (Hahn et al., 2019; Straathof et al., 2019).

When considering the parcellations dedicated to AVI, the regression analysis revealed that GA at birth is not consistently associated with any of the interlayer metrics considered here. In contrast, a positive significant effect was found for edge overlap across low-density levels for the whole-brain Myers parcellation, suggesting that by term age, the correspondence of edge architecture across the most consistent brain connections is higher in FT neonates. This may partly explain why functionality in PT neonates is altered but not absent and why distinct facets of an ability are differently affected (Pravia & Benny, 2020; Ream & Lehwald, 2018). Tracking assortativity along development may unveil its association with PT sequelae. Due to uncertainty in the selection of assortativity metrics (Noldus & Van Mieghem, 2015), we computed both Pearson or Spearman assortativity measures. Our findings from the exploratory stage argue against substantial deviations between the two. However, differences between both correlation coefficients seem to increase at higher density levels, which are more prone to influences from inter-individual differences and false positives.

### 4.4 Interhemispheric differences

Because reports of interhemispheric differences in AV tasks have come from studies on infants aged 4 months or older (Altvater-Mackensen & Grossmann, 2016; Ujiie et al., 2018), we expected brain asymmetries to be absent at term age. Our findings were mostly in line with this expectation, as both for the parcellation derived from the MMP atlas and the one including visual and auditory components only, main and interaction effects were generally absent in terms of global and local efficiency, and interlayer metrics. We interpret that the lateralized responses observed later in life likely depend on early stages of language development and conceptual knowledge, thus being absent directly after birth. However, our exploratory analysis with area under the curve as a summary measure hinted the presence of significant interhemispheric differences in the structural network when considering the MMP atlas. Such discrepancy with our pre-registered analysis, after which interhemispheric differences were generally absent, highlights the importance of selecting adequate approaches to account for cost-topology dependencies.

### 4.5 Exploratory analysis

When fixing a range of cost levels, effects within the selected threshold levels may remain occult (Ginestet et al., 2011). An exploratory regression analysis using the areas under the curve as integrated summary measures of global and local efficiency showed a similar pattern as our preregistered analysis using the MMP atlas. Specifically, a significant negative association between prematurity and global efficiency in the structural network. This is in contrast with the findings from the whole-brain Myers parcellation, according to which GA at birth is positively and negatively related to global and local efficiency in the structural connectome, respectively. Although this indicates a difference between networks comprising the entire brain and a subset of brain regions reported in the AVI literature, the effect of different parcellation characteristics was not contemplated. In this sense, when comparing the entire Myers parcellation and its auditory and visual components, our findings are not consistent.

Another step in our exploratory analysis showed that contrary to the structural connectome, functional connectivity strength was substantially larger in FT than in PT infants across all three parcellation schemes. We created functional connectomes using Pearson correlation, such that connectivity strength relates to the similarity of BOLD signals (statistical dependency) across the brain and over time (Bijsterbosch et al., 2017). This finding is consistent with other studies which show that prematurity is associated with altered or decreased functional connectivity (e.g., Ball et al., 2016; Brenner et al., 2021; Duerden et al., 2019; Smyser et al., 2016). Considering that altered FC predicts cognitive outcomes in PT individuals (e.g., He et al., 2018), the present findings contribute to the understanding of atypical AV-processing abilities in PT infants (e.g., Berdasco-Muñoz et al., 2019; Imafuku et al., 2019) even in the absence of altered network topology.

### 4.6 Limitations and methodological considerations

Limitations of the present study relate to different options for performing connectivity analysis (e.g., Bastiani et al., 2012; Centeno et al., 2022) and which integration and segregation metrics are used (Rubinov & Sporns, 2010). We encourage future research to follow different analytical pipelines with the aim of complementing the findings reported here (see Kristanto et al., 2024). Important analytical decisions include the tractography algorithm, the FC measure to extract time series, the treatment of negative correlation coefficients (Centeno et al., 2022; Hallquist & Hillary, 2019), as well as weighted (Batalle et al., 2017; Buchanan et al., 2020) or aggregated topology (Ginestet et al., 2011) and assortativity measures (Lim et al., 2019). The reported non-preregistered analysis using two additional parcellation schemes constitutes an attempt to reduce the noise introduced by heterogeneous methodological decision in the graph theory literature, but many others remain (Hallquist & Hillary, 2019). Although our findings on assortativity are consistent with the majority of the scientific literature, heterogeneous results have been linked to methodological differences (Fjell et al., 2017; Hahn et al., 2019; Huang & Ding, 2016), urging researchers to add to robustness by following different approaches. The choice of statistical models to test the proposed confirmatory hypotheses may limit our findings, in that excessively large models imply stricter corrections for multiple comparisons, potentially obscuring substantial effects. Including larger vPT and ePT subsamples may help overcome this issue by putting forward potentially stronger and robust between-group differences.

The majority of the current work was preregistered at https://osf.io/5px9e/. However, we incurred in small deviations from the preregistration because of unforeseen properties of the processed data: (1) the simulated null distributions to test the first confirmatory hypothesis (between group differences in network edge architecture) showed critical deviations from normality for the structural connectome at lower density levels. We reason that this is due to the higher robustness of structural connections (less variability across subjects: Hahn et al., 2019). Keeping only a very small proportion (0.05–0.10) of the most consistent edges in an already small network (52 nodes) led to the preponderant presence of the same edges irrespective of the resampling process and ultimately, to a subset of possible edge overlap scores. Considerably higher mean edge overlap scores at these density levels reinforce this notion (see Fig. A.1 in the Supplementary Material section). We argue that *p*-values estimated relative to such null distributions are misleading and, therefore, excluded in the present study. (2) Topology metrics resulted in reduced inter-individual variability and atypical inter-individual distributions at high density levels (see Fig. A.2 in the Supplementary Material section), especially for global efficiency. Such distributional properties are unsuitable for the regression analysis conducted here. We, therefore, excluded topology metrics at >0.5 density levels from the regression analysis.

## 5 Conclusion

This study aimed to investigate the association between PT birth and various aspects of functional and structural networks encompassing the entire brain, auditory and visual networks, and brain regions which have been reported in infant and adult neuroimaging studies to be engaged in AVI. The heterogeneity of our findings across parcellation schemes and outcome variables results in deviations and alignments with our working hypotheses. We showed that PT birth is associated with the edge architecture of a structural network across the entire brain and across ROIs extracted from the MMP atlas. The association between network properties in the latter and prematurity suggested an advantage for PT neonates, but could not be replicated in a parcellation encompassing visual and auditory networks only. We explored the correspondence between SC and FC by investigating assortativity in a multiplex network. We observed considerable correspondence between both connectivity types across parcellation schemes but found little evidence of associations with prematurity, mostly restricted to the whole-brain level. An exploratory analysis consistently showed differences in functional connectivity strength, with higher scores for FT neonates.

## Supplementary Material

Supplementary Material

## Data Availability

Data processing and statistical analysis scripts will be made publicly available at https://osf.io/5px9e/. Preprocessed data used in the present study can be accessed by registering to the Developing Human Connectome Project (dHCP); http://www.developingconnectome.org/project/. The dHCP administrators are in charge of evaluating requests and granting access.
