# Supplementary material for: Audiovisual integration and whole-brain networks in preterm and full-term neonates: A two-layer multiplex network perspective on structural and functional connectivity
Source: Imaging Neurosci (Camb). 2025 Oct 17;3:IMAG.a.928. doi: 10.1162/IMAG.a.928 (PMC12534712; doi:10.1162/IMAG.a.928)
Supplement: Supplementary Material [file IMAG.a.928_supp.pdf]

Supplementary material

Table A.1. Regions of interest and atlas used in the current study.

**Assigned label**	**Anatomical correspondence**	**Label in original atlas**	**Atlas**
V1	Visual area V1	V1_ROI	MMP atlas
V2	Visual area V2	V2_ROI	
V3	Visual area V3	V3_ROI	
V4	Visual area V4	V4_ROI	
InP	Intraparietal	IPS1_ROI	
		MIP_ROI	
		LIPd_ROI	
		AIP_ROI	
		IP0_ROI	
PIT	Posterior inferotemporal	FFC_ROI	
		PIT_ROI	
sPreM	Superior premotor area	6a_ROI	
		6d_ROI	
		FEF_ROI	
		55b_ROI	
iPreM	Inferior premotor area	PEF_ROI	
		6r_ROI	
		6v_ROI	
A1	Primary auditory area	A1	
A2	Auditory area	LBelt_ROI	
		PBelt_ROI	
		RI_ROI	
		MBelt_ROI	
pASS	Posterior associative area	STSvp_ROI	
		STSdp_ROI	
aASS	Anterior associative area	STSda	
		STSva	
psASS	Posterior superior associative area	A4_ROI	
		A5_ROI	
asASS	Anterior superior associative area	TA2_ROI	
		STGa_ROI	
aINS	Anterior insula	MI_ROI	
		AVI_ROI	
		AAIC_ROI	
TPOJ	Temporoparietaloccipital area	TPOJ1_ROI	
		TPOJ2_ROI	
		TPOJ3_ROI	
sIPC	Superior inferior parietal area	IP1_ROI	
		IP2_ROI	
iIPC	Inferior inferior parietal area	PGs_ROI	

		PFm_ROI	
lACC	Lower strip anterior cingulate cortex	33pr_ROI	
mACC	Middle strip anterior cingulate cortex	a24_ROI	
		p24_ROI	
		a24pr_ROI	
		p24pr_ROI	
sACC	Superior strip anterior cingulate cortex	a32pr_ROI	
		p32pr_ROI	
		d32_ROI	
		p32_ROI	
		s32_ROI	
lIFG	Lower strip inferior frontal cortex	44_ROI	
		45_ROI	
		47l_ROI	
sIFG	Superior strip inferior frontal cortex	IFJa_ROI	
		IFJp_ROI	
		IFSp_ROI	
		IFSa_ROI	
		p47r_ROI	
aMFG	Anterior medial frontal cortex	8C_ROI	
		8Av_ROI	
		8BL_ROI	
		8Ad_ROI	
		s6-8_ROI	
		i6-8_ROI	
		SFL_ROI	
pMFG	Posterior medial frontal cortex	9p_ROI	
		9a_ROI	
		p9-46v_ROI	
		a9-46v_ROI	
		46_ROI	
		9-46d_ROI	
SC	Superior colliculi	SC	Brainstem Navigator

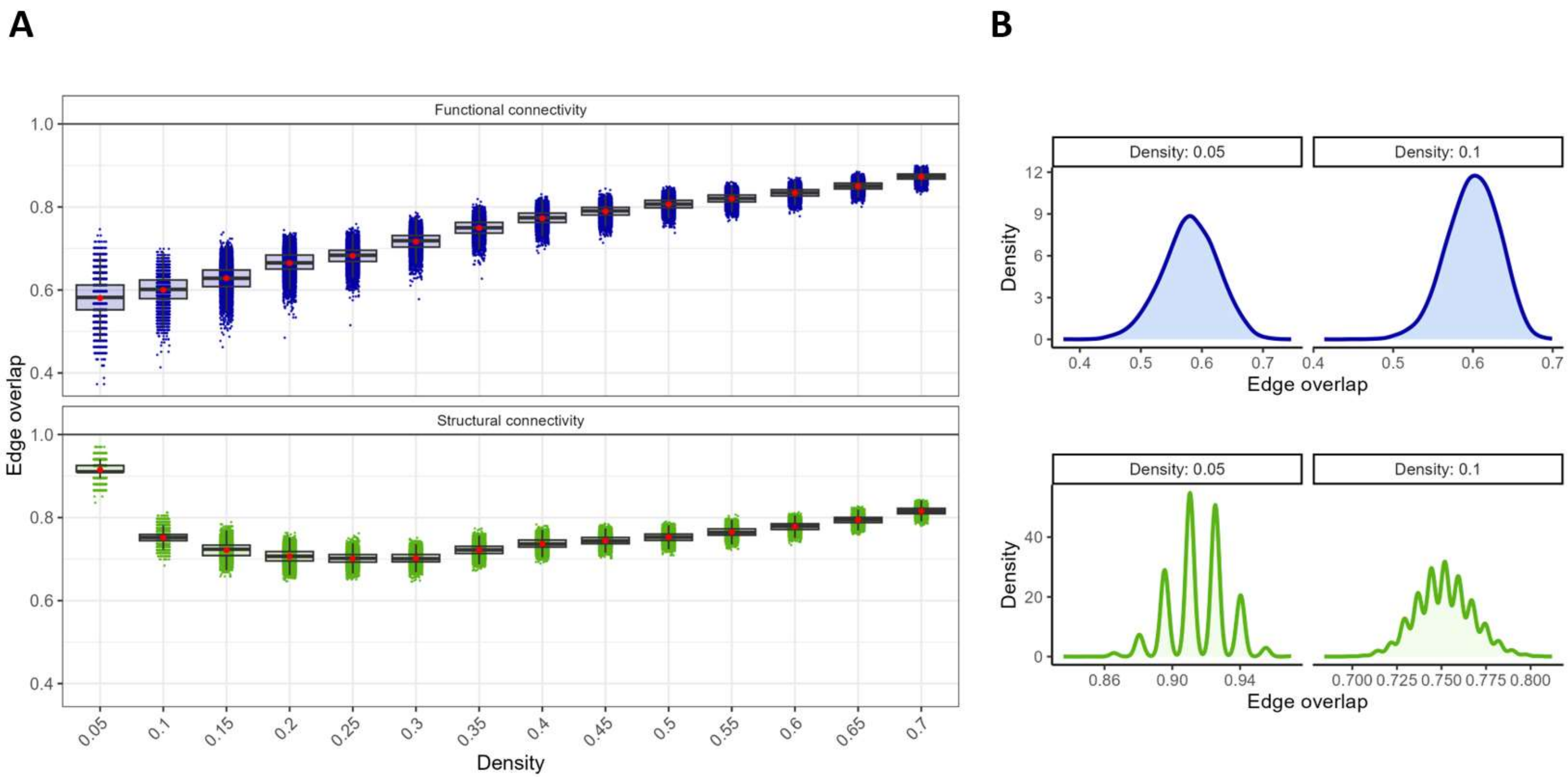

Fig A.1. Panel A: Boxplots of edge overlap scores between preterm and full-term groups computed along the permutation procedure (10000 iterations) for each density level and connectivity modality. Red dots indicate the mean score. Panel B: Density plots of the distributions in panel A at the two lowest density levels. Atypical shapes due to a small set of edge overlap values are observed for the structural connectome only.

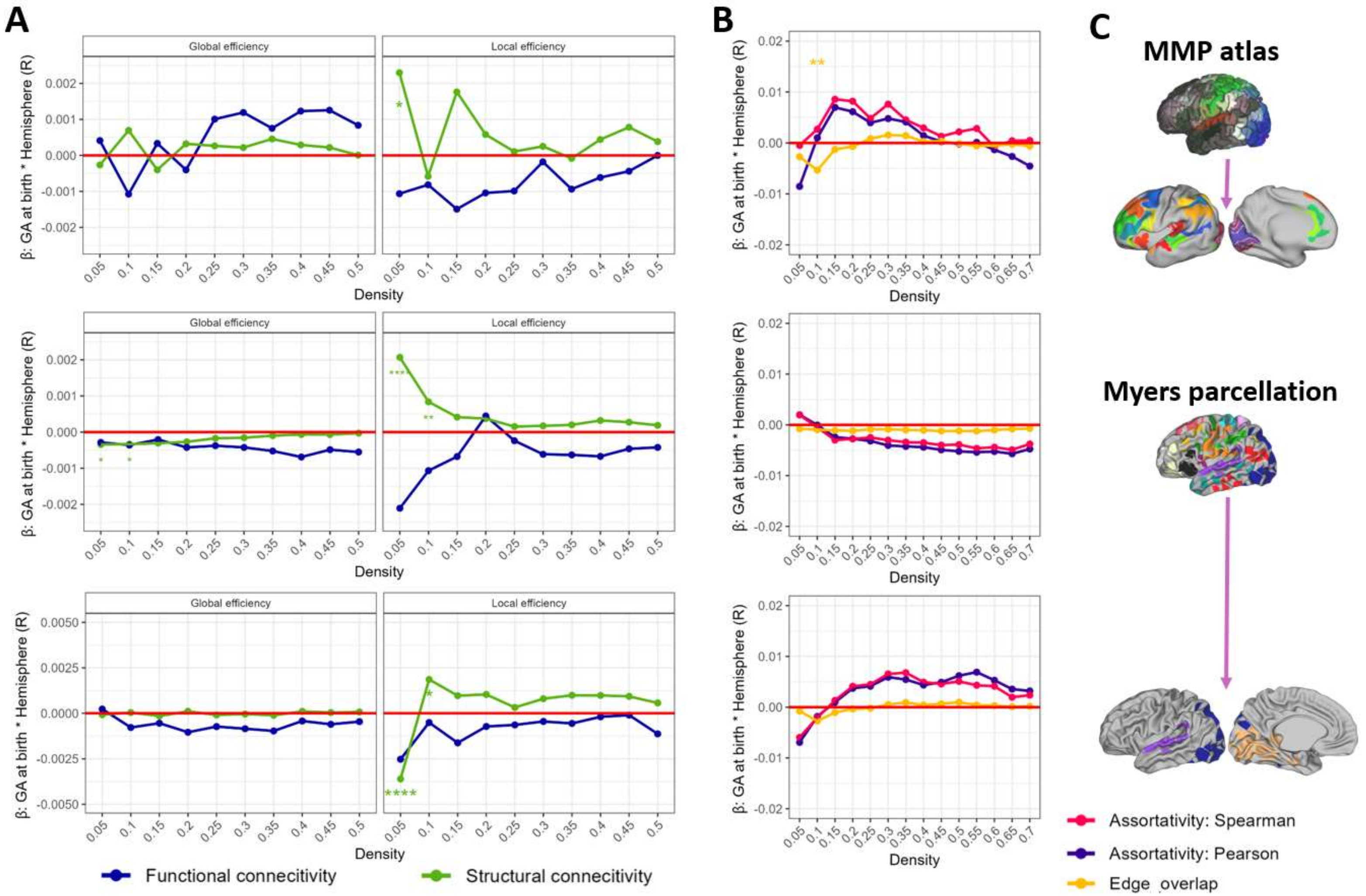
A
B
C
Global efficiency
Local efficiency
β: GA at birth * Hemisphere (R)
Density
Functional connecitivity
Structural connecitivity
MMP atlas
Myers parcellation
Assortativity: Spearman
Assortativity: Pearson
Edge overlap

Fig A.2. For each parcellation schemes (panel C), regression coefficients of interaction effects between gestational age at birth and brain hemisphere predict network global and local efficiency (panel A) and interlayer metrics (panel B). Significance levels after adjusting for multiple comparison: * ($p < .05$), ** ($p < .01$), *** ($p < .001$), **** ($p < .0001$).

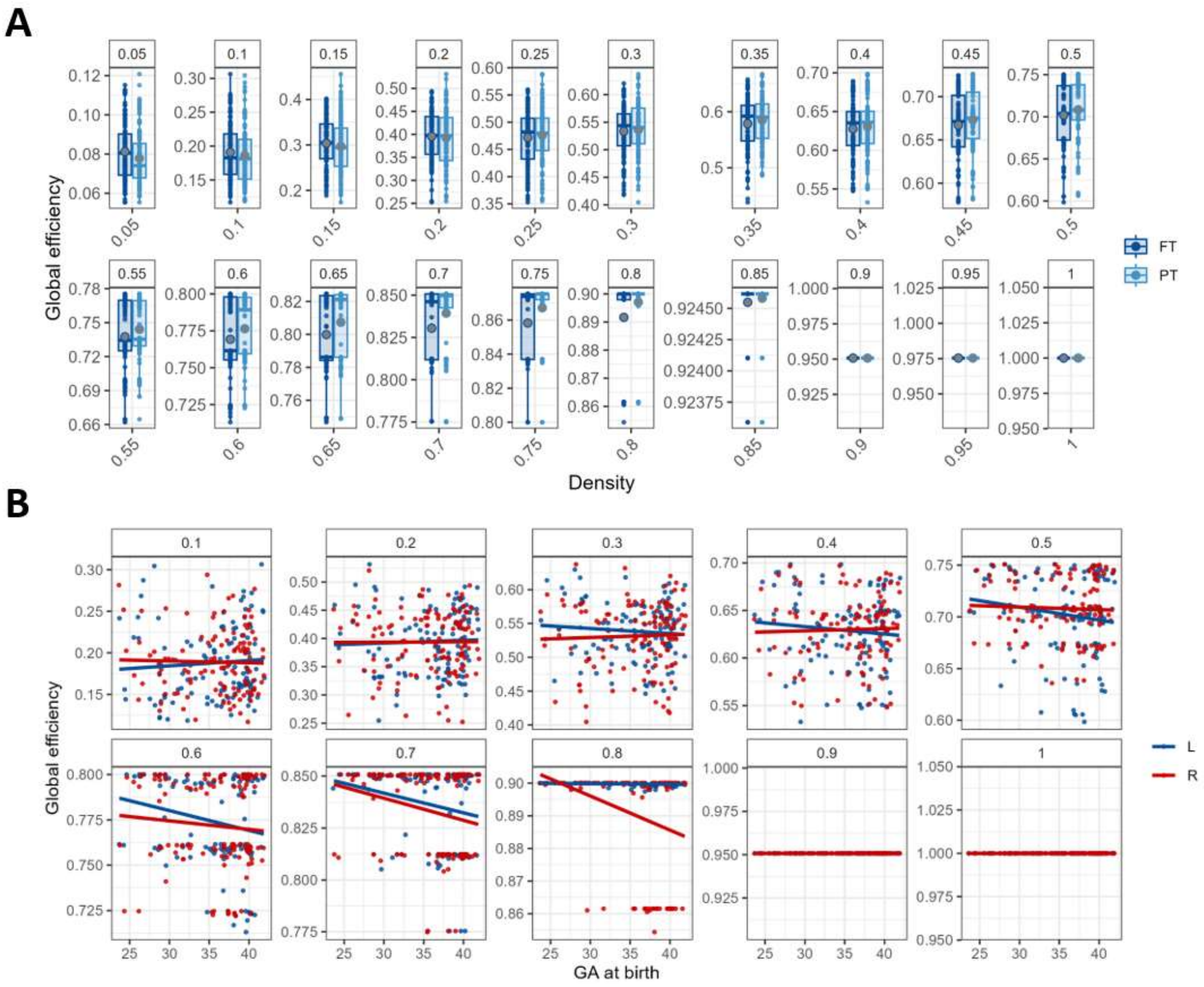
A
0.05
0.1
0.15
0.2
0.25
0.3
0.35
0.4
0.45
0.5
0.55
0.6
0.65
0.7
0.75
0.8
0.85
0.9
0.95
1
Global efficiency
Density
FT
PT
B
0.1
0.2
0.3
0.4
0.5
0.6
0.7
0.8
0.9
1
Global efficiency
GA at birth
L
R

Fig A.3. Global efficiency scores in the functional connectome are taken as example of distributional properties across density levels (0.05 - 1). Panel A: Global efficiency scores for full-term and preterm groups across density levels. Global efficiency scores approach 1 at high density levels, deviating from normal distributions and reducing inter-individual differences. Such distributional properties bias the usage of linear regression models for either brain hemisphere (panel B).
